# Tarin stimulates granulocyte growth in bone marrow cell cultures and minimizes immunosuppression by cyclo-phosphamide in mice

**DOI:** 10.1371/journal.pone.0206240

**Published:** 2018-11-07

**Authors:** Lyris A. D. Mérida, Érika B. A. Mattos, Anna C. N. T. F. Corrêa, Patricia R. Pereira, Vania M. F. Paschoalin, Maria F. B. Pinho, Mauricio A. Vericimo

**Affiliations:** 1 Department of Immunobiology, Biology Institute, Universidade Federal Fluminense (UFF), Rio de Janeiro, Brazil; 2 Department of Biochemistry, Chemistry Institute, Universidade Federal do Rio de Janeiro (UFRJ), Rio de Janeiro, Brazil; Temple University, UNITED STATES

## Abstract

Chemotherapeutic drugs, such as cyclophosphamide, cause severe immunosuppression and patients become susceptible to infections. Based on this, the immunomodulatory potential of tarin, a lectin from *Colocasia esculenta*, was evaluated in bone marrow cell cultures and in cyclophosphamide-immunosuppressed mice. Tarin promoted maintenance of hematopoietic progenitors and repopulation of Gr1 cells *in vitro* which was supported by *in vivo* results. In immunosuppressed mice, tarin increased bone marrow cell numbers and altered cell profile distribution by enhancing the frequency of Gr1^+^ progenitors, including Ly6-C^int^Ly6-G^lo^, and anticipating their proliferation/differentiation in mature cells, especially Ly6-C^lo^Ly6-G^hi^. Bone marrow cells harvested from tarin-treated immunosuppressed mice proliferated in response to GM-CSF or G-CSF *in vitro* and, the low numbers of bone marrow cells in the G0 phase, combined with a high number cells undergoing apoptosis confirmed that tarin promoted a faster and intense proliferation/differentiation, even in the presence of CY-induced toxicity. As a result, tarin minimized leukopenia in immunosuppressed mice promoting a faster recovery of peripheral leucocytes and protected erythroid bone marrow cells from CY-cytotoxicity in a dose-dependent manner. Data suggest that tarin could be considered a potential adjuvant to decrease leukopenia and possibly ameliorate anemia, if carefully evaluated in human cancer cell lineages and in clinical trials.

## Introduction

Chemotherapeutic drugs, such as cyclophosphamide (CY), cause severe lymph and myelosuppression, resulting that >10% of the population undergoing chemotherapy becomes susceptible to infections [1]. Chemotherapy-induced leukopenia is the major cause of viral, bacterial and fungal infections that are often life-threatening. Besides the threat these infections represent to patients’ lives, often resulting in reductions in the chemotherapy dose intensity that may impact oncologic outcomes, they are also a major burden to public health, since they typically require hospitalization, resulting in high medical costs [2]. Although supportive therapy with growth factors minimizes leukopenia and the risk of infection [3, 4], those cytokines currently in use only stimulate specific cell lineages, requiring a combination of drugs, which increases drug treatment expenditure. Low-cost substances with immunomodulatory activities may be used as adjuvants to prevent opportunistic infection as a strategic treatment for the amelioration of chemotherapy-induced immunosuppression.

Lectins are proteins or glycoproteins, derived from plants and other organisms, that can be obtained at a relatively low cost and display clinical significance and therapeutic potential, due to their anti-HIV, anti-tumoral, antimicrobial, anti-inflammatory and anti-nociceptive activities [5, 6].

Our research group successfully purified to homogeneity (>90%) a lectin from taro (*Colocasia esculenta*), named tarin, using a simple, replicable, fast, and low-cost procedure [7]. Tarin was fully characterized, revealing a highly stable molecule to a wide range of pH and temperatures and displaying the ability to specifically bind to high-mannose and complex N-glycans [8, 9]. Moreover, purified tarin exhibits mitogenic activity on splenocytes and total bone marrow cells [7], especially B-lymphocytes, suggesting that it could be useful for alleviating immunosuppression in certain types of cancer. Granulocyte loss, particularly neutrophils, is characteristic of chemotherapy-induced immunosuppression and is the main factor for high infection susceptibility. Despite all these features, few lectins have been tested as potential drugs to revert immunosuppression [10–13].

In the present study, the potential therapeutic of tarin as an immunomodulatory agent was evaluated in bone marrow cell cultures and in CY-immunosuppressed mice. Tarin allowed the maintenance of hemopoietic progenitor cells favoring the growth of granulocytes *in vitro* and *in vivo*. In addition, tarin minimized leukopenia in immunosuppressed mice promoting a faster recovery of peripheral leucocytes and protected erythroid bone marrow cells from CY-cytotoxicity in a dose-dependent manner, suggesting that tarin might be useful as an immunomodulatory adjuvant in therapeutic regimens.

## Materials and methods

### Animals

Adult male C57BL/6 mice (8 to 12 weeks old) were provided by the Laboratory Animal Nucleus (NAL), located at the Biology Institute of the Universidade Federal Fluminense (UFF), Brazil. The animals were maintained under conventional environmental conditions with exhaust fans, at a room temperature of 23–25°C, fed with Nuvilab CR-1 chow (Nuvital Nutrientes S/A, Colombo, BRA) and acidified water *ad libitum*. Research protocol was approved by the Animal Experimentation Ethics Committee (CEPA) at NAL-UFF, under number 670/2016.

### *Colocasia esculenta* corms and tarin purification

*Colocasia esculenta* (L.) Schott corms were manually chosen and purchased from a local market in Rio de Janeiro, Brazil. The crude taro extract (CTE) was obtained according to Roy, Banerjee, Majumder, & Das [14] and was stored at –20°C until tarin purification steps. Tarin purification was performed according to the protocol described previously by Pereira *et al*. [7], by affinity chromatography through a Cibacron Blue 3GA (Sigma-Aldrich Co, MO, USA) column. Protein concentrations of the tarin fractions were estimated by the Lowry method [15], using bovine serum albumin (BSA) (Sigma-Aldrich Co) at 1mg/mL for the standard curve.

### Bone marrow cell suspensions and culture conditions

The animals were anesthetized with an overdose of 40mg/kg xylasin and 200mg/Kg ketamin and were sacrificed by cervical dislocation. Bone marrow (BM) cells were obtained by percolating the femurs with sterile phosphate buffered saline (PBS). Cell suspensions were washed twice in PBS, centrifuged at 258 × *g* at 4°C on centrifuge PR-2 (IEC–Co Inc., TN, USA). Pellet cells were subjected to osmotic shock by the addition of a hypotonic solution (5 x diluted PBS with distilled water) to eliminate erythrocytes. A cell sample was diluted in Turk’s solution, transferred to a Neubauer chamber (Labor Optik, Lancing, UK), and counted under an optical Olympus BX41 microscope (Olympus America Inc., NY, USA).

Cells were cultured (2 × 10^4^ cells/mL) in RPMI-1640 media (Sigma-Aldrich Co), supplemented with 10% fetal calf serum (FCS), 2 mM L-glutamin, 5 x 10^−5^ M 2-mercaptoethanol and 20 μg/mL gentamicin, in the presence or absence of 20 μg/mL tarin, at 37°C in a humidified atmosphere containing 5% CO_2_, for 19 days. Medium were replaced every 5 days, and cell samples were collected in established days to analyses. Cells harvested from the cultures on days 0, 3, 6, 10, 13, 16, and 19, were transferred to glass slides by centrifugation (284 × *g* for 10 min at room temperature) using a Cytopro 7620 centrifuge (WESCOR Inc, UT, USA). Cells were analyzed after staining by the May-Grunwald-Giemsa method and at least 100 cells were counted under optical microscopy (Olympus BX41) to determine relative cell numbers [16]. Photomicrographs of the cultures were acquired under an inverted-phase microscope Zeiss Telaval 31 (Carl Zeiss Co., Oberkochen, DEU).

### Clonogenic assays

BM cells were obtained on day 4 from distinct mice groups: **CY**–mice immunosuppressed with CY 300 mg/kg (Genuxal) (Baxter Hospitalar Ltda, MG, BRA); **CY+Tarin**—CY-immunosuppressed mice treated concomitantly with 200 μg tarin on day 0; **Tarin**—mice treated with 200 μg tarin on the same day or **Control**—mice inoculated with saline. Cells at 2×10^5^ were plated in double layer soft-agar prepared as described by Heyworth and Spooncer [17]. The bottom layer was prepared at a 0.4% final agar concentration in Iscove’s medium (Sigma-Aldrich Co) with 20% FBS, plated in 34-mm TPP tissue culture dishes (Sigma-Aldrich Co). The upper layer containing the cells (0.33% final agar concentration) was supplemented either with 20% supernatants of WEHI and MM3 cells or rHu-G-CSF at 60 μg/plate. Each assay was carried out in duplicate and cultures were incubated at 37°C in a humidified atmosphere containing 5% CO2. The colonies (>50 cells) and clusters (<50 cells) were quantified after 7 days of culture under an inverted microscope.

Filgrastine (Blau Farmacêutica S.A., SP, Brazil) was used as source of recombinant human granulocyte colony-stimulating factor (rHu-G-CSF). The cell lines WeHi 3B and MM3 were obtained from the Rio de Janeiro Cell Bank (APABCAM, RJ, Brazil) and their supernatants were also used as a source of IL-3 and GM-CSF, respectively.

### Bone marrow cell proliferation and death evaluation

To study the effects of tarin administration on the cell cycle and apoptosis, BM cells were obtained from mice on day 4 after as follows: **CY**–mice immunosuppressed with CY 300 mg/kg; **CY+Tarin**—CY-immunosuppressed mice treated concomitantly with 200 μg tarin on day 0; **Tarin** -mice treated with 200 μg tarin on the same day or **Control**—mice inoculated with saline. The assays were performed according to the protocol established by Riccardi and Nicoletti [18] with few modifications. Cells at 2 x 10^6^ were washed in PBS, centrifuged at 200 x *g* for 5 min at 4°C, suspended in 500 μL PBS and fixed by adding 4.5 mL of 70% (v/v) of cold ethanol. Cell suspensions were centrifuged at 400 x *g* for 5 min at 4°C and the pellets were washed in 5 mL of PBS. After centrifugation at the same conditions, cells were suspended in 500 μL of PBS and 500 μL of DNA extraction buffer (192 mL of 0.2 M Na_2_HPO_4_ with 8 mL of 0.1% Triton X-100) were added to each sample. Cell suspensions were incubated for 5 min at room temperature, centrifuged at 400 x *g* for 5 min, suspended in 1 mL of DNA staining solution (200 μg of propidium iodide in 10 mL of PBS) and incubated for 2 hours at room temperature until analyzed on a BD FACSCALIBUR Cytometer (BD Bioscience, NJ, USA). Data were processed with the aid of the FlowJo, LLC software (Oregon, USA).

### Flow cytometry cell analysis and monoclonal antibodies

Cells freshly obtained from BM and from the BM cultures were counted by the exclusion test using Trypan blue to determine cell viability [19]. Cells suspensions were incubated with 200 μL of blocking solution (3% FCS + 10% normal mouse serum in PBS and 0.001% sodium azide) for 15 min at 4°C to prevent non-specific antibody bindings. Cells collected from cultures were washed with PBS, centrifuged at 700 x g for 10 min at 4°C, and then, incubated with biotin anti-mouse Ly-6G/Ly-6C (Gr-1) antibody (Bio Legend Inc., CA, USA) for 30 min at 4°C. The primary antibody was revealed with streptavidin-allophicocyanin (SAV-APC) (Bio Legend Inc.) for 30 min at 4°C, cells were washed with PBS and centrifuged at 700 x *g* for 7 min at 4 ^o^C. Pellet cells were fixed in 200 μL of PBS containing 1% formaldehyde to further analyses. BM cell suspensions were washed with PBS, centrifuged at 700 x *g* for 10 min at 4°C, and incubated for 30 min at 4°C with the follow conjugated anti-mouse antibodies: FITC anti-CD45, Phycoerythrin anti-Ly6-G, Percep Cy5.5 anti-Ly6-C or anti-c-Kit, (Bio Legend Inc.). Biotin anti-Ly-6G/Ly-6C (Gr-1) or anti-CD11b were revealed with SAV-APC when required. Cells were washed with PBS, centrifuged at 700 x *g* for 7 min at 4°C and fixed with PBS containing 1% formaldehyde. Cells were gated according to the expression of CD45 (≥98%). Cell analyses were performed on a BD Accuri C6 Flow Cytometer (BD Bioscience) or BD SCALIBUR Cytometer. Fluorescence intensity was analyzed with the aid of Summit 4.3 software (Beckman Coulter Inc., CA, USA).

### Peripheral blood cell analysis in immunosuppressed mice treated by tarin

To investigate the effects of tarin on peripheral blood cells of immunosuppressed mice, the following protocol was established. Mice were divided in four groups (n = 4), which intraperitoneally received: *i)* sterile physiological saline (**Control**); *ii)* 200 μg tarin on days 0, 2, and 5 (**Tarin**); *iii)* 300 mg/kg cyclophosphamide on day 0 (**CY300**); and *iv)* 300 mg/kg cyclophosphamide on day 0, followed by 200 μg tarin on days 0, 2, and 5 (**CY300 + Tarin**). Blood cell parameters from each group were analyzed during and after treatments, as described in the following.

Anesthetized mice from each group were bled on days 0, 2, 5, and 7 by the retro-orbital plexus, with the aid of a Pasteur pipette, to determine the number of circulating leukocytes and the Hematocrit. Blood sample were transferred to microfuge tubes containing 50μL of heparin (25 UI/mL). Collected blood was diluted 1:100 in Turk’s solution, to eliminate erythrocytes, and the number of peripheral blood leukocytes (PBLs) was counted in a Neubauer chamber. For hematocrit measurements, blood was transferred to capillary glass tubes previously treated with heparin 50UI/mL, one of the ends was sealed and the tubes were centrifuged at 715 × *g*, 4°C for 10 min. Hematocrits were determined by the ratio between the total column height (erythrocytes + plasma) and the erythrocyte column height.

### Analysis of tarin effects on bone marrow cells of immunosuppressed mice

To evaluate the effects of tarin on BM cells from CY-immunosuppressed mice, appropriate assays were conducted as described on previous section, with modifications. In three independent experiments, CY-immunosuppressed mice received 200 μg tarin intraperitoneally: *i*) after 2 days; *ii*) after 3 days and *iii*) after 4 days. The animals were euthanized 24h after tarin exposure. In other set of experiments, CY-immunosuppressed mice received a unique intraperitoneal dose of tarin right after CY inoculation on day 0, and mice were euthanized on days 4, 5, 7 and 9. Mice BM cells from each set of experimental group was removed and cell suspensions were properly prepared and evaluated by flow cytometry and clonogenic assays, as described previously.

### Analysis of tarin effects on bone marrow erythroid cells of immunosuppressed mice

To study the effects of tarin administration on erythroid lineage cells of mice submitted to cytotoxicity caused by distinct CY doses, animals were divided into 5 groups. Each group received intraperitoneally: *i)* CY at 50 mg/kg on day 0 (**CY50**); *ii)* CY at 300 mg/kg on day 0 (**CY300**); *iii)* CY at 50 mg/kg on day 0 followed by 200 μg tarin on days 0, 2 and 5 (**CY50 + Tarin**); *iv)* CY at 300 mg/kg on day 0 followed by 200 μg tarin on days 0, 2 and 5 (**CY300 + Tarin**); *vi)* sterile physiological saline (**Control**).

On the 6th day after treatment, mice from each group (n = 4) were sacrificed after anesthesia by cervical dislocation, the BMs were removed and cell suspensions prepared as previously described, however, in this assays, erythrocytes were not eliminated. Cell samples were smeared on glass slides, dried overnight at room temperature and stained by Leishman staining [20]. The occurrence of nucleated erythrocytes was quantified at an optical Olympus BX41 microscope.

### Statistical analyses

Multiple comparison analyses were performed by one-way or two-way ANOVA followed by Tukey *post-hoc* test [21]. Significance was considered when *p*<0.05, as determined by the GraphPad Prism 7.0 Software (GraphPad Software Inc., CA, USA).

## Results

### Tarin exhibited protective and stimulatory effect on mice bone marrow cells *in vitro*

**Fig 1:**
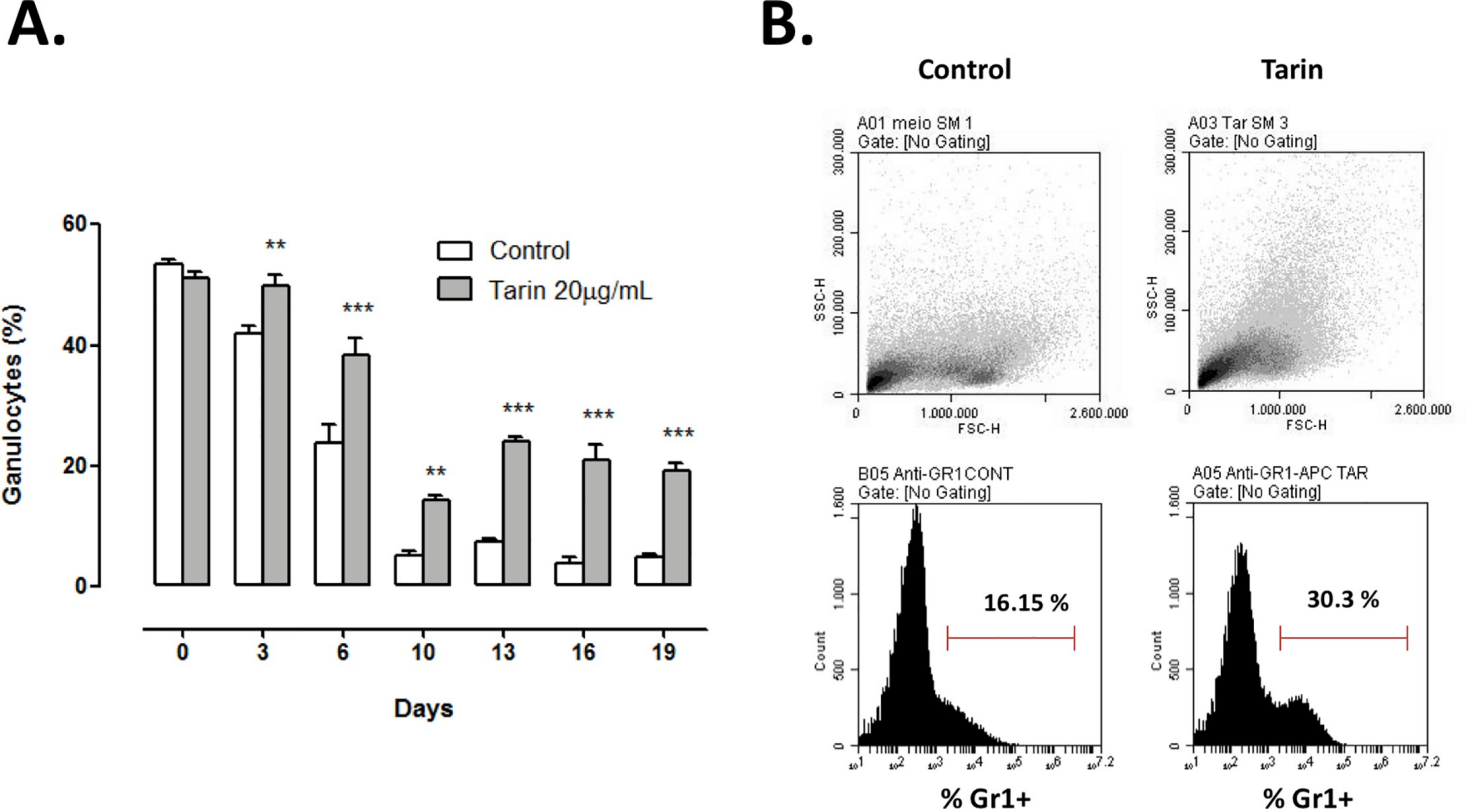
Protective and stimulatory effects of tarin in mouse bone marrow cell cultures. **(A)** Granulocyte frequency on days 3, 6, 10, 13, 16, and 19 from the mouse BM cells cultured with 20 μg/mL tarin. Cultures without tarin addition were used as control. **(B)** Cell distribution profile of the BM cells on 6^th^ day of culture with and without tarin addition (*top panels*). Frequency of Gr1^+^ cells on the cultures from BM cells on 6^th^ day of culture are indicated on the histogram plots (*bottom panels*). Data were obtained from n = 3 experiments. *indicates significance level. *** *p*<0.001 and ** *p*< 0.01, compared to control.

To investigate the immunomodulatory potential of tarin, mice BM cells were cultured in the presence of tarin and the granulocytes was evaluated by cytospin and flow cytometry. Tarin exhibited protective and stimulatory effects on BM cell cultures, as indicated by the maintenance of the granulocyte ratio to total cells, particularly from day 10 to day 19 (Fig 1A). Control cell cultures in the absence of tarin, at 3 and 6 days, showed a decrease in granulocyte numbers of 20% and 55%, respectively, whereas in the cell cultures, after tarin addition, the number of granulocytes displayed a discrete reduction of 5% and 25%, at the same time periods (Fig 1A). From day 10 to day 19, a drastic reduction in granulocyte counts was observed in the control cultures, where the remaining cells reached 5% of total cells. On the other side, cultures that received tarin were able to maintain a ratio around 45% of granulocytes during the same time period (Fig 1A).

To confirm the stimulatory effects of tarin on myeloid lineage cells, a flow cytometry analysis was performed on the 6^th^ day of culture. Dot plots (Fig 1B, *top panel*) showed a variation in cell distribution profiles of BM cells cultured in the presence or absence of tarin. Tarin maintained a consistent high percentage of Gr1^+^ cells (30.3 ± 5.3%) when compared to non-tarin stimulated cells (16.1 ± 2.1%) (Fig 1B, *bottom panel*).

**Fig 2:**
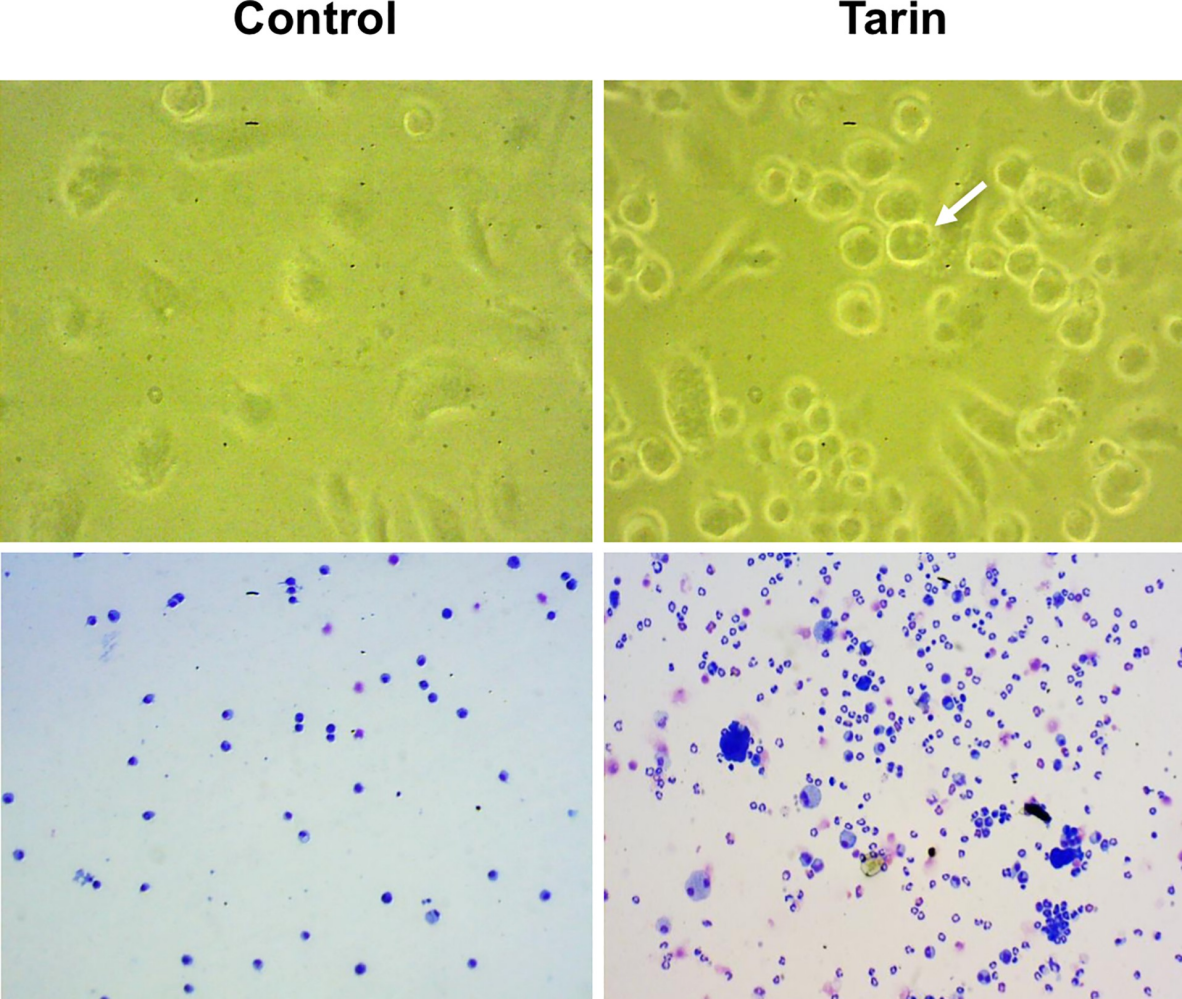
Morphological characteristics of bone marrow cells cultured with tarin. BM cell cultures incubated with tarin 20 μg/mL (Tarin) and without tarin (Control) for 6 days (*top panels*) and 19 days (*bottom panels*). The white arrow indicates a typical nucleus of a myeloid cell in development. The bottom panel shows granulocytic lineage cells in distinct developmental stages in tarin-treated cells (right-hand side) or the control group (left-hand side). Photomicrographs were acquired by an inverted-phase microscopy under 400x magnification (*top panel*) or by visualization of cytosmears, stained by May-Grunwald Giemsa, using optical microscopy under 200x magnification (*bottom panel*).

Tarin-treated cells revealed the presence of many fibroblast-like adherent cells and refringent rounded cells with a ring nucleus in the cultures after 6 days, in contrast with that observed in cultures grown in the absence of tarin, where the predominance of subcellular elements (debris) were observed during same time period (Fig 2, *top panel*). In addition, cells harvested at day 19 presented morphological characteristics of mature granulocytes and also precursor cells of the granulocytic lineage in distinct developmental stages (Fig 2, *bottom panel*), suggesting that maintenance/differentiation of such cells can occur *in vitro* induced by tarin *stimuli*.

### Tarin protective effect in immunosuppressed mice

**Fig 3:**
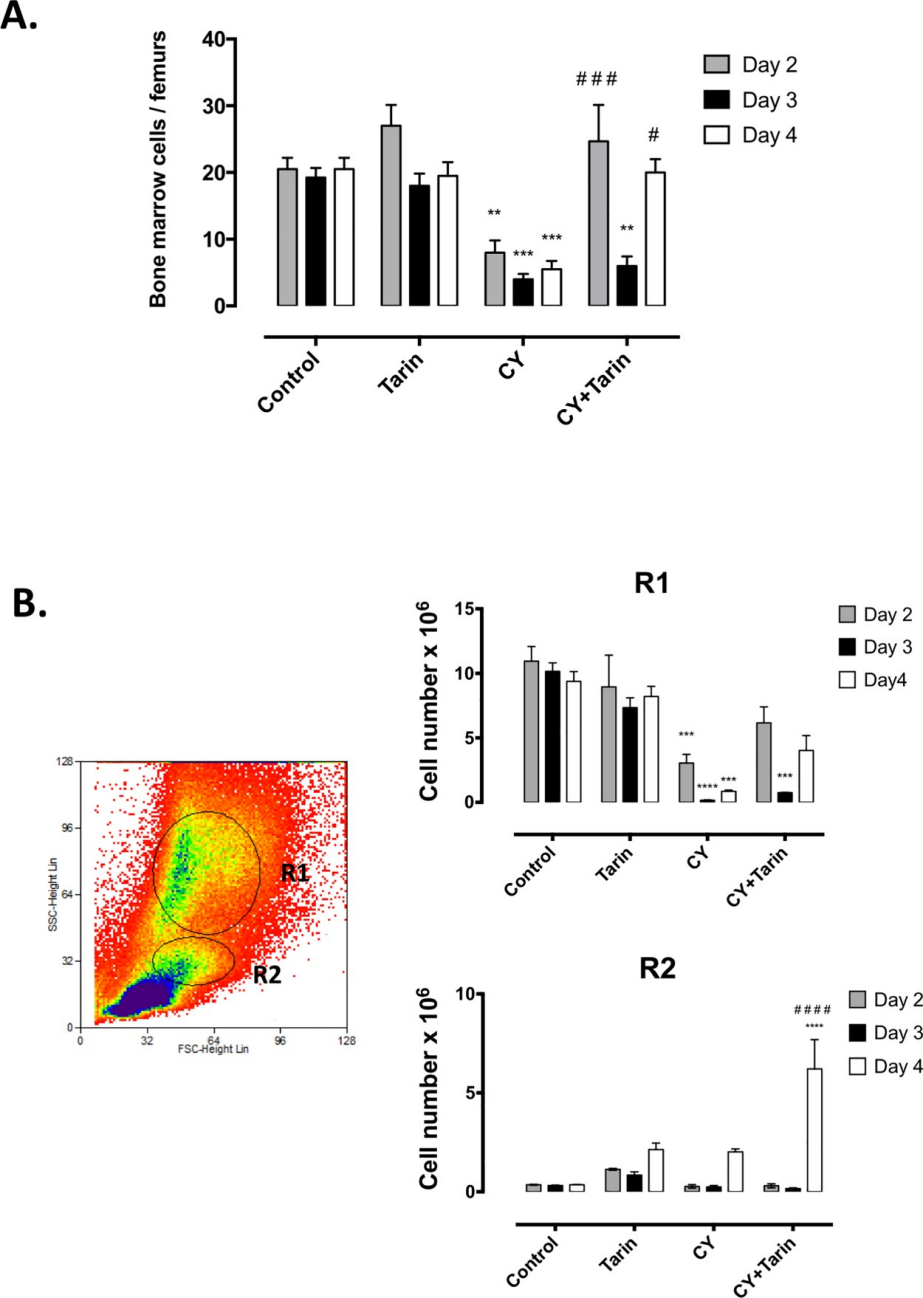
Tarin effects on bone marrow cell numbers. **(A)** Total BM cell numbers from: **CY**- CY-immunosuppressed mice; **CY+Tarin**—CY- immunosuppressed mice treated with 200 μg tarin; **Tarin**—mice treated with 200 μg tarin or **Control**–mice inoculated with saline. Mice BM cells were evaluated after 24h tarin treatment on days 2, 3 or 4. **(B)** Dot plots on the left-hand panel represent cell distribution profile (size *vs* granularity) from healthy BM cells by flow cytometry. The R1 region corresponds to granulocytic lineage cells, while R2 corresponds to mononuclear/blastic cells based on cell size and granularity. Right-hand panels show the total number of cells in R1 and R2 from mice BM treated by the aforementioned protocol. ****p<0.0001, ***p<0.001 and ** *p*<0.01, compared to control. #*p*<0.05, ###*p*< 0.001 or ####*p*< 0.0001 compared to CY group.

Tarin was able to stimulate myeloid BM cells *in loco* in CY-immunosuppressed mice exposed to tarin on days 2, 3 or 4 (Fig 3A). Tarin administration to immunosuppressed mice caused an increase in the number of BM cells when administered on day 2 or 4, after CY-challenge, recovering control cell levels on such days (Fig 3A). Although the absolute number of cells in the granulocytic region (R1) was not significantly different comparing both groups on the analyzed days, a clear increasing trend was observed (Fig 3B, *top panel*). On the other hand, the absolute number of cell in the mono/blastic cells region (R2) strongly increased in tarin-treated immunosuppressed mice on day 4 (Fig 3B, *bottom panel*).

**Fig 4:**
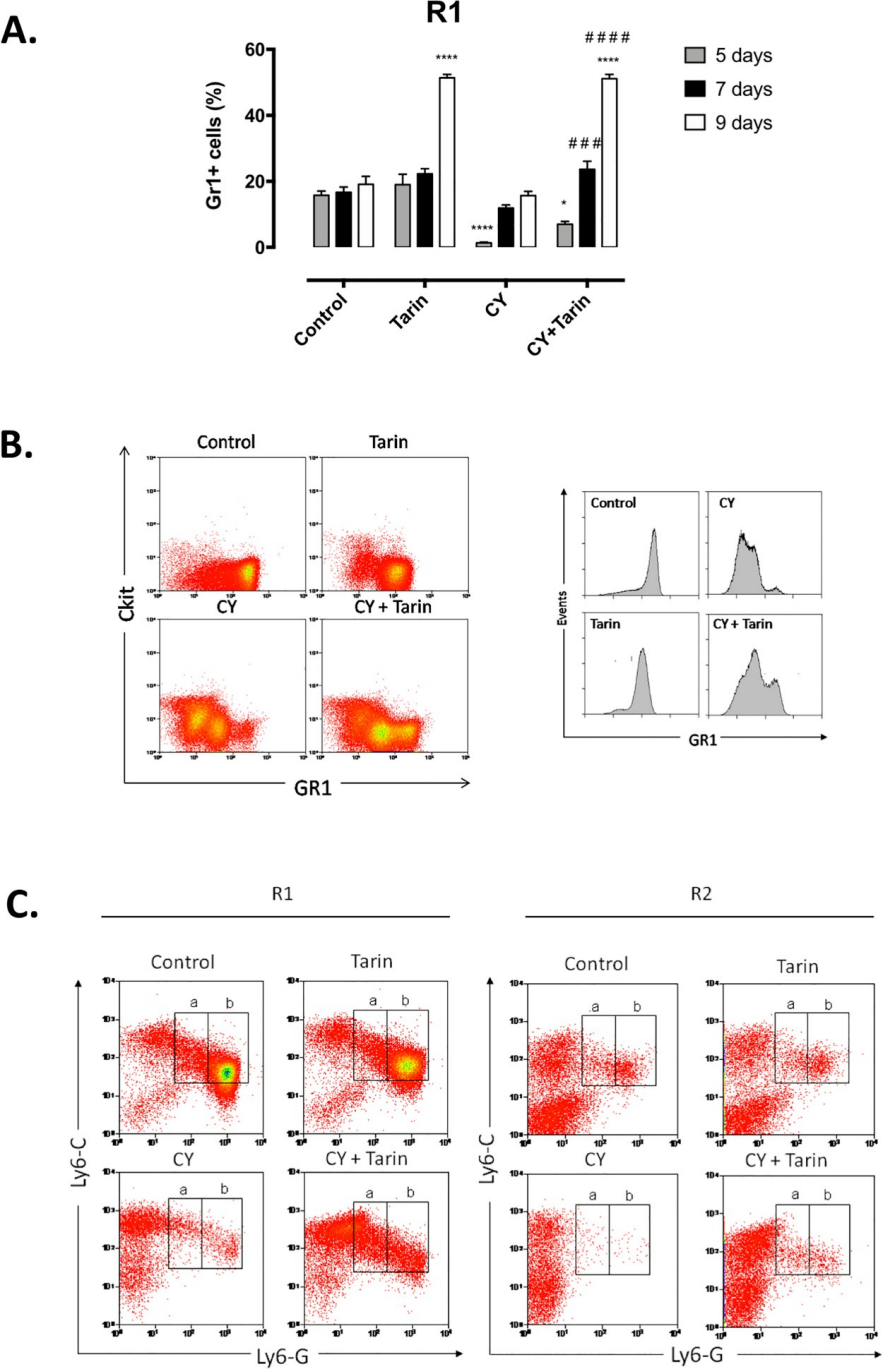
Frequency of granulocytic cells in the bone marrow of CY-immunosuppressed mice treated with tarin. **(A)** Frequency of Gr1^+^ cells in the BM of CY-immunosuppressed mice treated with a unique dose of 200 μg tarin right after CY-challenge (day 0). Analyses were performed 5, 7 and 9 days after tarin inoculation and cells corresponding to R1 (indicated on Fig 3) were evaluated. ****p<0.0001 or *p<0.05 compared to control group. ###*p*<0.001 or ####*p*<0.0001 compared to CY group. **(B)** Dot plot representing Gr1 and c-kit expression on R1 of BM cells from each mice group, as defined in Fig 3 legend, on the 4^th^ day after tarin administration (*top panel*). Histograms in the bottom panel show Gr1 expression intensity in the same region. **(C)** Dot plot of Ly6-G and Ly6-C cell markers expression on R1 and R2 of BM cells from distinct mice groups. Ly6-C^int^Ly6-G^lo^ cells are shown in “a” rectangle and Ly6-C^lo^Ly6-G^hi^ cells are shown in “b” rectangle. Data are representative of three independent experiments (n = 3).

In the other set of experiments, mice concomitantly received CY and tarin and were euthanized after 4, 5, 7 and 9 days. In CY-immunosuppressed mice, the BM cell profile showed a decrease in the granulocytic and an increase in mono/blast cell regions at day 4 (S1 Fig). However, when associated to tarin, an increase in the granulocytic and mono/blast cell regions was observed compared to control mice (S1 Fig). An increment in the Gr1^+^ cells frequency in immunosuppressed mice exposed to tarin for 7 or 9 days was observed (Fig 4A). Tarin exposure alone caused an increase in the frequency of Gr1^+^ cells in the bone marrow, evidenced just after 9 days (Fig 4A). Differences in Gr1 frequency and expression were observed as early as the 4^th^ post-exposure day to tarin, as displayed in the dot plot of the Gr1^+^ cell population (Fig 4B). A higher frequency of myeloid progenitors Gr1^int^c-kit^lo^ was observed in tarin-treated immunosuppressed mice, corresponding to 13% of total BM cells, while corresponding to 8% of total BM cells in CY-immunosuppressed mice (Fig 4B, *top panel*). In addition, the persistent presence of granulocytic cells Gr1^hi^c-kit^-^ was observed only in tarin-treated immunosuppressed mice, corresponding to 8.5% of total BM cells. Such cells were rare in CY-immunosuppressed mice (1.5% of total BM cells). Histograms of Gr1 expression intensity obtained from all experimental groups are presented in Fig 4B, bottom panel.

Further analysis of BM cells from CY-immunosuppressed mice at the granulocytic region (R1), indicated that tarin caused an increase in Ly6-C^lo^Ly6-G^hi^ granulocytes and in the Ly6-C^int^Ly6-G^lo^ cell population (Fig 4C, *left-hand panel*). Ly6-C^lo^ Ly6-G^hi^ cells represented 25% of BM cells in control mice and tarin-inoculated mice (Fig 4C, *left-hand panels*, b). In CY-immunosuppressed mice, such cells drastically decreased to 0.72% of total BM cells, after 4 days of CY-injection. However, in tarin-treated CY-Immunosuppressed mice, the number of Ly6-C^lo^Ly6-G^hi^ cells increased about 6-fold, reaching 4.3% of BM cells on the 4^th^ day (Fig 4C, *left-hand panels*, b). In addition, Ly6-C^int^Ly6-G^low^ cells reached 10% of BM cells, while it represented 1.7% in CY-immunosuppressed mice not treated with tarin (Fig 4C, *right-hand panel*, *a*). Both cell populations were detected in mono/blast cells region (R2) only in tarin-treated CY-immunosuppressed mice (Fig 4C, *right-hand panel*).

**Fig 5:**
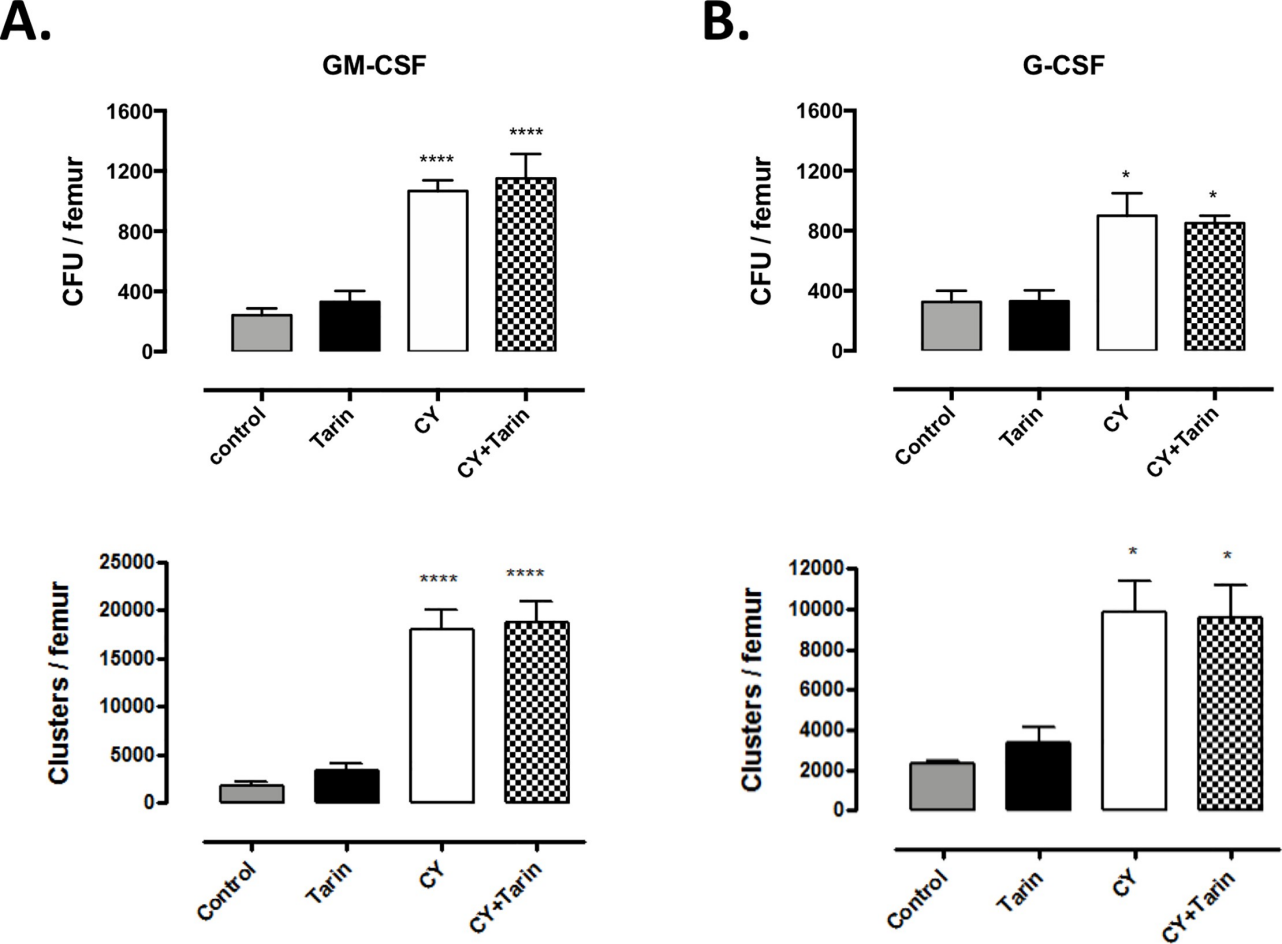
Clonogenic potential of bone marrow myeloid progenitors. Number of colony-forming units (CFU) and clusters in soft-agar culture of BM cells from: **CY**–CY-immunosuppressed mice; **CY+Tarin**—CY-immunosuppressed mice treated concomitantly with 200μg tarin on day 0; **Tarin**—mice treated with 200 μg tarin on the same day or **Control**—mice inoculated with saline. BM cells were collected on day 4 and plated with GM-CSF or G-CSF stimuli. Responses to GM-CSF **(A)** and to G-CSF **(B)** were analyzed on the 7^th^ day. Results are expressed as the mean values and standard errors of three independent experiments. *p<0.05, ****p<0.0001 compared to the control group.

**Fig 6:**
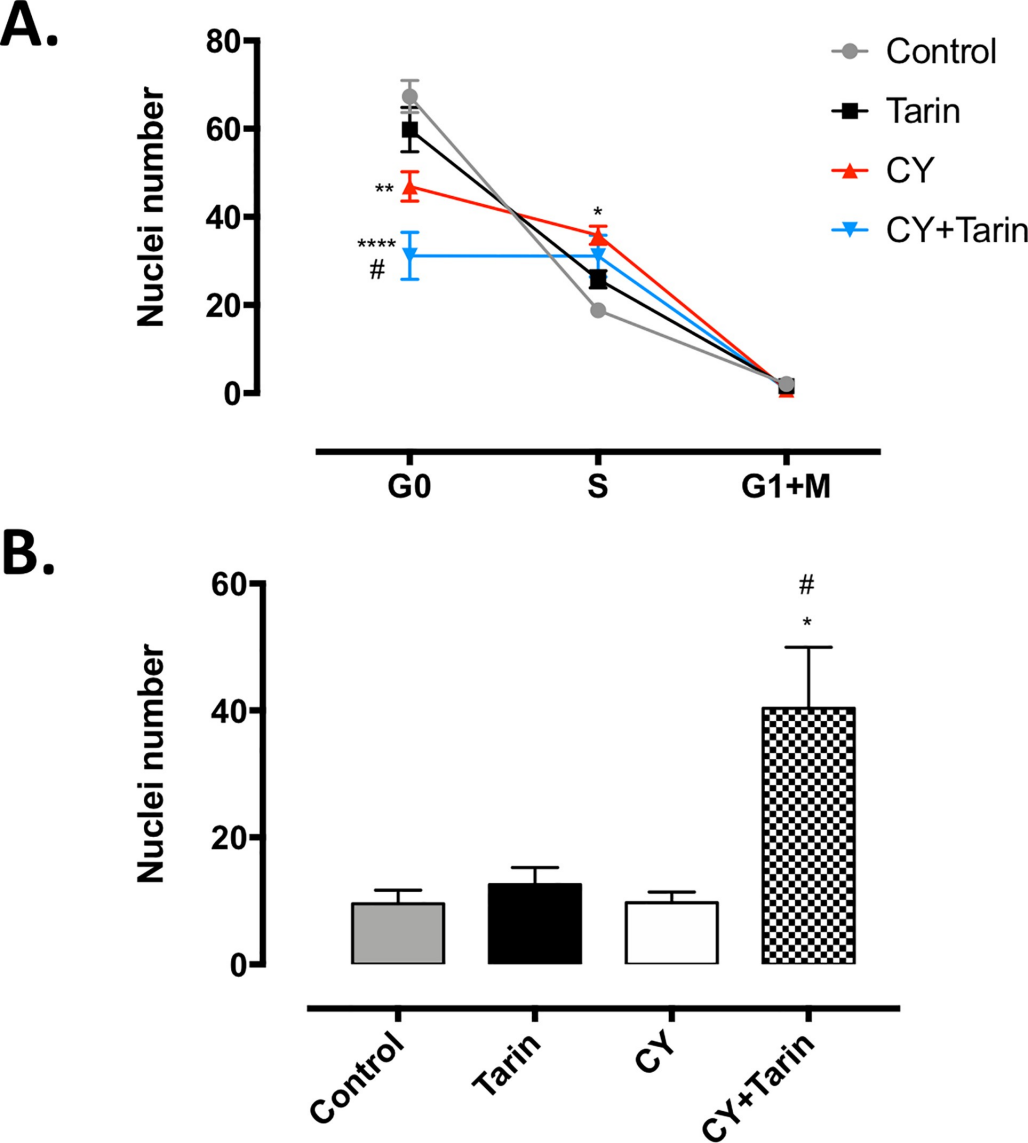
Tarin effects on bone marrow cell proliferation and death. Cell cycle **(A)** and apoptosis **(B)** analyses of BM cells from: **CY**–CY-immunosuppressed mice; **CY+Tarin**—CY-immunosuppressed mice treated concomitantly with 200 μg tarin on day 0; **Tarin**—mice treated with 200 μg tarin on the same day or **Control**—mice inoculated with saline. BM cells were evaluated by flow cytometry on day 4. *p< 0.05, **p< 0.01 and **** *p*<0.0001 compared to control group. #*p*< 0.05 compared to CY group.

To determine if BM cells harvested from tarin-treated immunosuppressed mice after 4 days would be able to proliferate *in vitro* in response to GM-CSF or G-CSF, colonies and cluster counts were determined in soft-agar culture after 7 days. Immunosuppressed and tarin-treated immunosuppressed mice exhibited a higher number of colonies and clusters compared to the control mice but no difference was observed between them (Fig 5). On the other hand, the number of BM cells in the G0 phase decreased (Fig 6A) and the number of cells undergoing apoptosis increased in immunosuppressed mice exposed to tarin for 4 days (Fig 6B).

**Fig 7:**
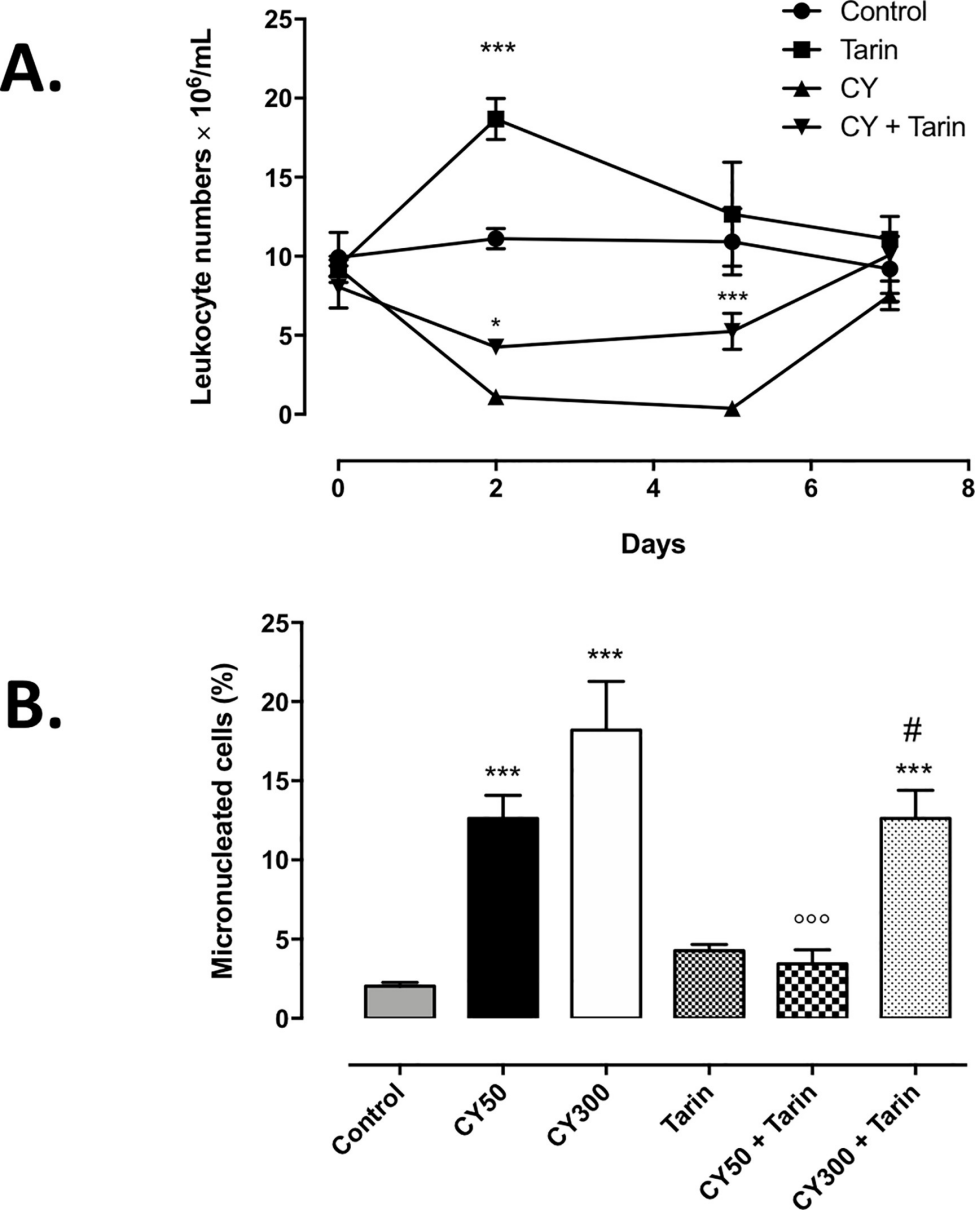
Tarin reduces cytotoxic effects in CY-immunosuppressed mice. **(A)** Leukocytes number in peripheral mice blood treated with: **CY**–CY-immunosuppressed mice; **CY + Tarin**–CY-immunosuppressed mice treated with 200 μg tarin on day 0, 2 and 5; **Tarin**–mice treated with 200 μg tarin on days 0, 2, and 5; and **Control**–animals inoculated with saline. Blood samples were collected on days 0, 2, 5, and 7. ***p<0.001 represents Tarin *vs* Control comparison on day 2, and CY + Tarin *vs* CY on day 5. *p< 0.05 compares CY + Tarin to CY on day 2. **(B)** Occurrence of micronuclei in BM erythroid cells from: **CY300**—mice immunosuppressed with 300 mg/kg CY; **CY50**—mice immunosuppressed with 50 mg/kg; **CY300 + Tarin**—mice immunosuppressed with 300 mg/kg and treated with 200 μg tarin and **CY50 + Tarin**—mice immunosuppressed with 50 mg/kg and treated with 200 μg tarin. ***p<0.001 compared to control;°°°*p*< 0.001 compared **CY50** to **CY50+Tarin**, and #*p*< 0.05 compared **CY300** to **CY300+Tarin**.

The CY-injection caused a drastic reduction in the number of circulating leukocytes soon after drug administration (Fig 7A). However, after tarin administration to CY-immunosuppressed animals, the drops in leukocytes were minimized on days 2 and 5 (4.51 ± 0.9 x 10^6^ and 5.25 ± 0.7 x 10^6^ cells, respectively) and returned to basal levels on the 7^th^ day (Fig 7A). Interestingly, control mice that received tarin only exhibited an increase in circulating leukocytes from day 0 until day 2 (19.12 ± 1.5 x 10^6^ cells) (Fig 7A). No difference was observed in hematocrit levels between groups (S2 Fig).

Cyclophosphamide caused a dose-dependent increase in the frequency of micronucleated erythrocytes in BM *in situ*, in accordance to the high cytotoxicity of this drug. On the other hand, tarin administration protected erythroid BM cells from the cytotoxic effects of CY, particularly at CY 50 mg/kg, decreasing the frequency of micronucleated erythrocyte cells to basal levels. A minor protective tarin effect was evidenced at CY 300 mg/kg (Fig 7B).

## Discussion

Immunomodulatory molecules act on immunological components resulting in stimulation, suppression or modulation of the immune system [22]. Plant lectins, including tarin, fulfill the requirements to become potential immunomodulatory agents based on their ability to bind to specific carbohydrates found on the surface of immunological cells [22, 23]. Herein, tarin minimized the decrease of granulocytes in culture in the absence of growth factors. Tarin in mouse BM cell cultures attenuated the decrease in granulocyte frequency, maintaining cells even during 19 days of culture. We have demonstrated that tarin binds to glycan chains found in LeY (CD174) and H2 (CD173) surface molecules highly expressed (> 60%) in CD34^+^ hematopoietic progenitor cells and in peripheral blood granulocytes [8, 24, 25]. It is possible that, the interaction of tarin with CD174 and/or CD173 molecules could favor the maintenance of progenitor cells in culture, as well as the production of granulocytes at high frequency from days 10 to 19. Moreover, photomicrography of mice BM cells culture indicated the presence of fibroblast-like cells and refringent rounded cells, rare in the non-treated culture, suggesting a putative activity of tarin on stromal cells. Further studies are necessary to determine if tarin acts directly on hematopoietic cells in a carbohydrate-dependent manner or if it acts on stromal cells triggering the release of growth or survival factors that could produce the aforementioned results. A synergic action should also be considered.

These *in vitro* effects have been described for other lectins belonging to tarin family, the GNA-related lectins, such as those found in banana, artocarpin, garlic, and dolichos. These lectins are able to promote the maintenance of human and murine cord blood C34^+^ cells, *in vivo* and *in vitro* [11, 12]. Studies have demonstrated the interaction of these lectins with stromal and CD34^+^ cells in a carbohydrate-dependent manner and the subsequent reduction of reactive oxygen species (ROS) level as a putative molecular mechanism for the maintenance of CD34^+^ cells [11, 12]. Another member of the GNA-related family, the NTL lectin, promoted the preservation of stem/multilineage hematopoietic progenitors *in vitro* and also stimulated their expansion *ex vivo* [26].

Tarin *in vitro* stimulatory and protective effects on progenitor hematopoietic cells were supported by *in vivo* results using the CY-immunosuppressed mice model. The effects of CY on mice reproduced those observed in patients under chemotherapy, where a strong leukopenia is observed [4]. Tarin inoculated in CY-immunosuppressed mice was able to increase total BM cells and alter the BM cell distribution profile, indicating the proliferation/differentiation activities of progenitor cells. In fact, both granulocytes and their progenitors in BM cells were present at a higher frequency than that observed in immunosuppressed animals not inoculated with tarin. In addition, an enhancement of mono/blastic cells was observed, supporting tarin potential action on the maintenance of hematopoietic progenitor cells. Tarin effect on myeloid cells became evident as early as day 4 after tarin administration, as evidenced by the increase in cells expressing high levels of Gr1 marker, which was rare on immunosuppressed mice not treated by tarin. Tarin seems to maintain progenitor cells and stimulate a faster repopulation of Gr1^hi^ cells *in vivo*. Since the Gr1 marker can be expressed in other cell types, including monocytes, macrophages and dendritic cells [27], the Ly6-G cell surface marker was evaluated. The presence of Ly6-C^lo^Ly6-G^hi^ mature granulocytes and granulocytic precursor Ly6-C^int^Ly6-G^lo^ cells, in the BM of tarin-treated CY-immunosuppressed mice, reinforce tarin potential to stimulate differentiation.

BM cell proliferation in response to GM-CSF and G-CSF in CY-immunosuppressed mice and tarin-treated CY-immunosuppressed mice, was quite similar in clusters and colony numbers on the days analyzed. Considering the higher CY toxicological effect on day 3 and the tarin effect on granulocytes at different cellular developmental stages, it is possible that the effects of tarin inoculation may be evidenced in early days and/or in clonogenic cell-type composition. Cell cycle and apoptosis analysis of BM cells from tarin-treated CY-immunosuppressed mice confirmed tarin ability to stimulate cell proliferation. The elevated number of cells under apoptosis in CY-immunosuppressed mice treated with tarin could reflect CY-toxicity combined with tarin stimulation of cell proliferation/differentiation in high levels. Thus, data suggests that tarin could promote faster repopulation and renewal of myeloid cells in emergency.

Regarding that particularly neutrophils play a crucial role in fighting infection [4], tarin may be regarded as an immunostimulatory molecule candidate for the recovery of the immunosuppression state. Since tarin also exhibits proliferative effects on total spleen and BM cells, especially B lymphocytes [7], the use of this molecule to avoid or minimize chemotherapy immunosuppression side effects was considered and carefully evaluated in the murine models herein. *In vivo* experiments confirmed that tarin inoculation attenuated CY toxicological effects, maintaining the number of PBLs higher than in non-treated immunosuppressed mice. Moreover, a proliferative response was induced in animals treated only by tarin, evidenced by the increase in the number of PBL, which peaked 2-fold after the second day of tarin treatment. Tarin administration also decreased the number of *in vivo* micronucleated erythrocytes, but did not prevent or attenuate the decrease in hematocrit levels in CY-animals, indicating a protective effect on erythroid progenitors, but probably not on their differentiation. Perhaps, additional signaling factors other than tarin would be necessary to induce erythrocyte differentiation.

Based on the obtained results, it could be inferred that tarin administration in immunosuppressed mice could protect hematopoietic progenitors, especially from granulocytic, and, possibly, erythroid lineages, anticipating proliferation/differentiation and consequent repopulation of BM and the release of novel leucocytes to peripheral blood.

The application of tarin as an adjuvant molecule to minimize CY effects should be considered in tumor-bearing mice models since tumor environments stimulate the abnormal development of Gr1^+^CD11b^+^ myeloid cells, giving rise to a myeloid-derived suppressor cell (MDSC) population. MDSCs facilitate tumor proliferation and migration through the production of suppressive factors, including COX-2 activation and prostaglandin E2 (PGE2) synthesis, with consequent suppression of T-lymphocytes, dendritic and natural killer (NK) cells [27–29]. Data presented herein are not enough to evaluate if the increase in granulocytic cells would interfere in antitumoral responses in a murine model or if T-lymphocytes responsiveness would be affected by tarin. However, Kundu et al. [30] have already studied the effect of tarin in tumor-bearing mice and demonstrated that tarin is able to decrease the proliferation of breast and prostate cancer cell lines, and completely block the migration of a highly metastic breast cancer. Metastasis in mice was attributed to anti-inflammatory tarin effects, via down-regulation of both prostaglandin E2 synthesis and mRNA levels of cyclooxygenase (COX) 1 and 2 [30]. In lung cancer models, tarin decreased metastasis by leading to an increase in the levels of TNF-alpha, IL-6, and IL-12 cytokines [30]. COX inhibitor agents have been extensively used in clinical trials to decrease breast, prostate and lung cancer proliferation, growth and migration by preventing the suppression of NK, dendritic and T cells caused by PGE2 enhancement [6, 31]. Based on this, it is possible that tarin effects on myeloid cells population described in this study may not impair antitumoral/antimetastatic responses. However, further studies should be carried out in order to characterize the myeloid populations stimulated by tarin, and determine myeloid cells effect on T cells responsiveness and, consequently, in tumor progression and metastasis in murine models.

Considering that chemotherapeutic drugs, including cyclophosphamide, can cause severe lymph and myelosuppression, and that over 10% of patients become susceptible to infections, tarin could be regarded as a promising immunomodulatory adjuvant molecule in chemotherapeutic regimens. Although the results presented here were obtained in a murine model by evaluating *in vitro*, *in vivo* and *ex vivo* tarin effects, for tarin, to be considered as a potential adjuvant, it should be carefully evaluated in human cancer cell lineages and in clinical trials.

## Conclusions

Tarin exhibits potential immunomodulatory properties, with the ability to protect granulocytic progenitor cells and promote their *in vitro* repopulation. In an immunosuppressed murine model, tarin led to the increased total BM cells and altered the BM cell profile distribution, enhancing the frequency of granulocytic progenitors (Ly6-C^int^Ly6-G^lo^), anticipating their proliferation/differentiation in mature cells, especially Ly6-C^lo^Ly6-G^hi^, while also possibly protecting erythroid progenitors, preventing their death. These effects resulted in leukopenia minimization in immunosuppressed mice treated by tarin, promoting a faster recovery of blood leukocytes. As a future perspective, the potential benefic effects of tarin administration could be explored as a chemotherapy adjuvant to treat anemia, leukopenia and sensitizing erythroid progenitor cells to erythropoietin.

## Supporting information

S1 Fig BM cells distribution profile. Size and granularity parameters of BM cells from: **CY**–CY-immunosuppressed mice; **CY+Tarin**—CY-immunosuppressed mice treated concomitantly with 200 μg Tarin on day 0; **Tarin**—mice treated with 200 μg tarin on the same day or **Control**—mice inoculated with saline. BM cells were evaluated by flow cytometry on day 4. Dot plots are representative of cell distribution profile of each group. A frequency of cells in granulocytic, mono/blastic and lymphocytes gate were expressed as means ± standard deviation of three independent experiments (n = 3). ***p< 0.001 and ****p<0.0001 compared to Control. # *p*< 0.05 compared to CY.
(TIFF)

S2 Fig Hematocrit analysis. Blood samples were collected on days 0, 2, 5, and 7 from groups: **CY**–CY-immunosuppressed mice on day 0; **CY + Tarin**–CY-immunosuppressed mice treated with 200 μg tarin on day 0, 2 and 5; **Tarin**–mice treated with 200 μg tarin on days 0, 2, and 5; and **Control**–mice inoculated with saline. Results are expressed as means ± standard deviation of three independent experiments (n = 3).
(TIFF)
