# Supplementary figures and images for: Tarin stimulates granulocyte growth in bone marrow cell cultures and minimizes immunosuppression by cyclo-phosphamide in mice
Source: PLoS One. 2018 Nov 7;13(11):e0206240. doi: 10.1371/journal.pone.0206240 (PMC6221300; doi:10.1371/journal.pone.0206240)

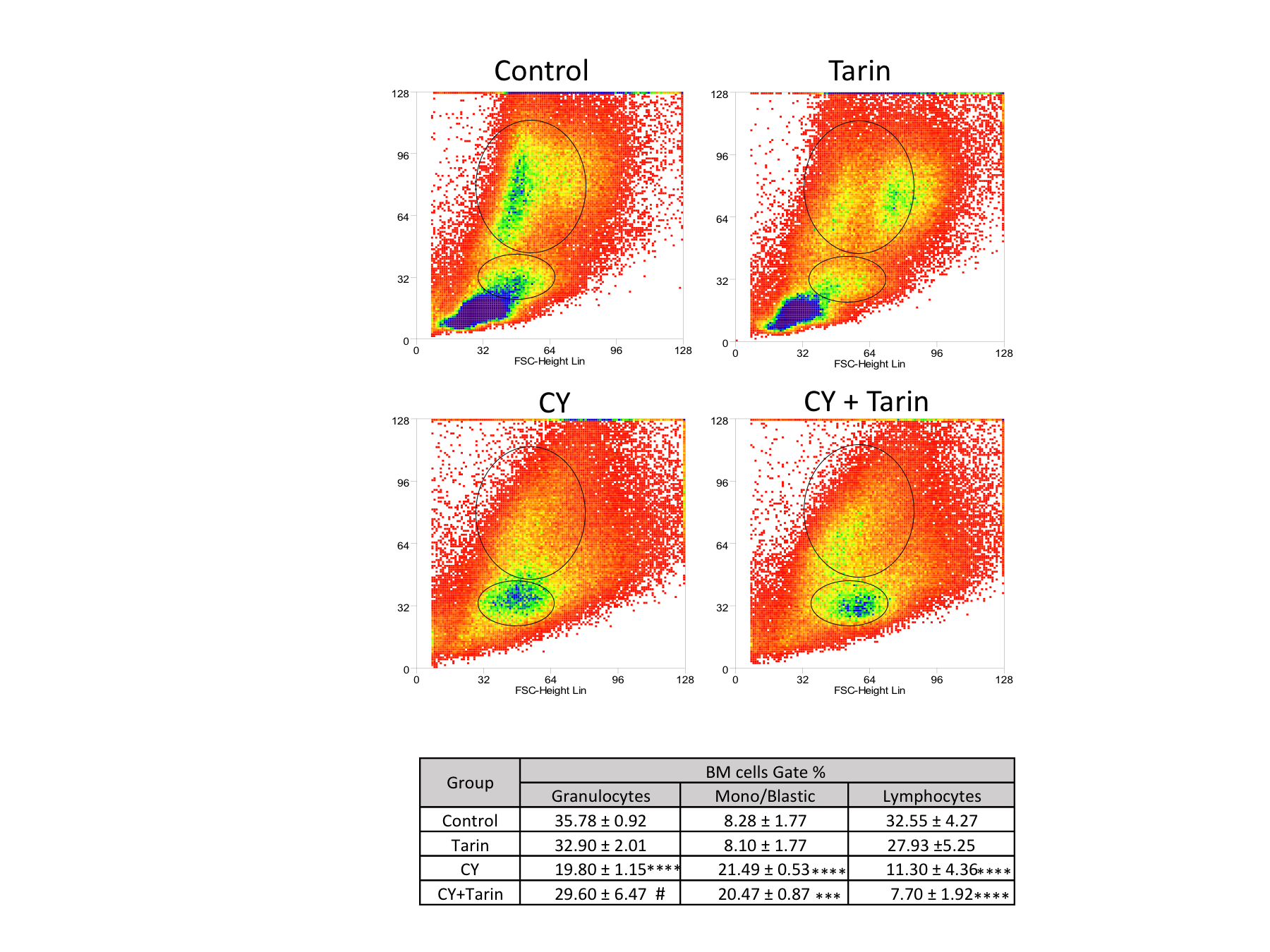

Supplement: S1 Fig — Size and granularity parameters of BM cells from: CY–CY-immunosuppressed mice; CY+Tarin—CY-immunosuppressed mice treated concomitantly with 200 μg Tarin on day 0; Tarin—mice treated with 200 μg tarin on the same day or Control—mice inoculated with saline. BM cells were evaluated by flow cytometry on day 4. Dot plots are representative of cell distribution profile of each group. A frequency of cells in granulocytic, mono/blastic and lymphocytes gate were expressed as means ± standard deviation of three independent experiments (n = 3). ***p< 0.001 and ****p<0.0001 compared to Control. # p< 0.05 compared to CY. (TIFF) [file pone.0206240.s001.tiff]

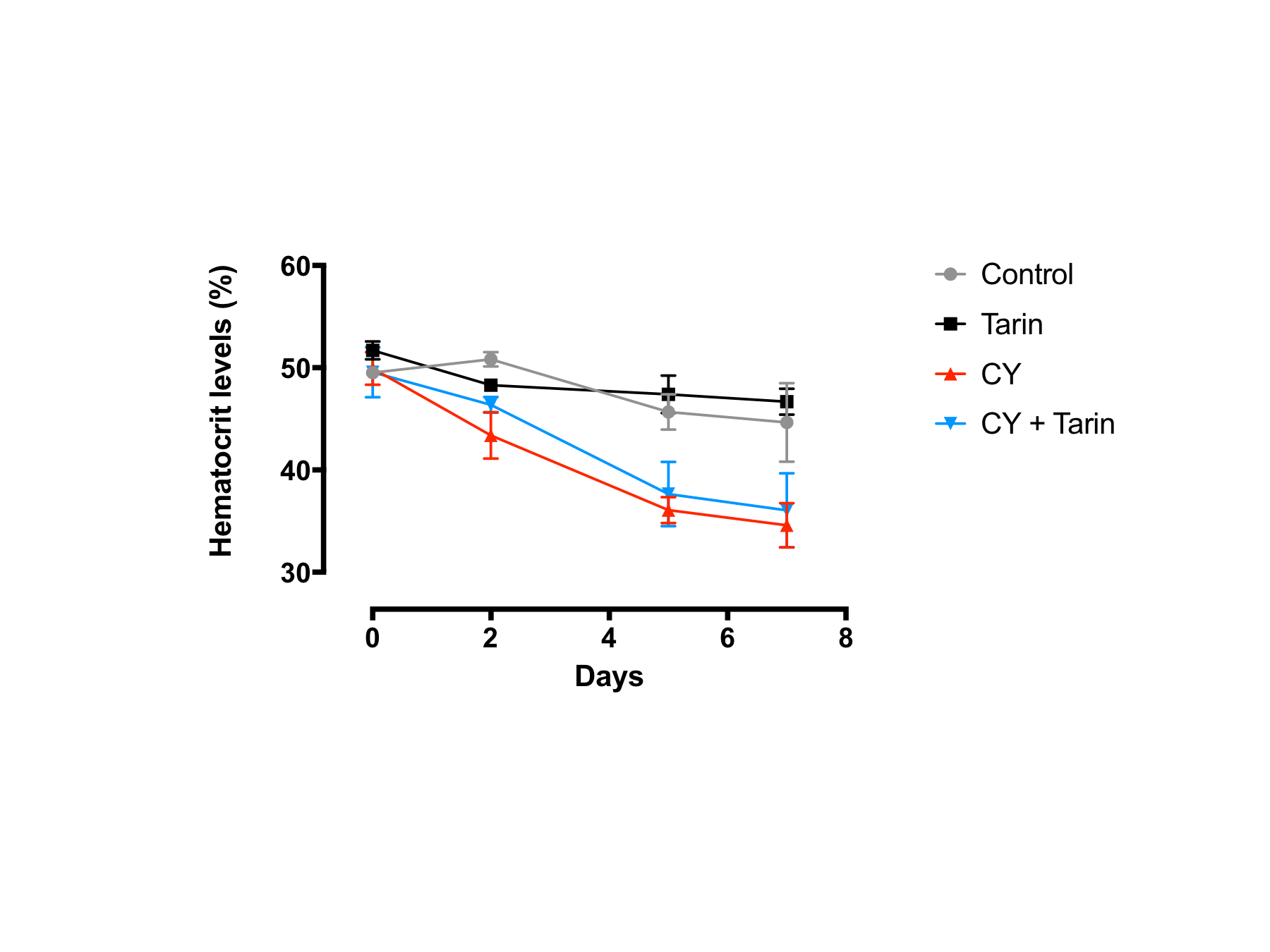

Supplement: S2 Fig — Blood samples were collected on days 0, 2, 5, and 7 from groups: CY–CY-immunosuppressed mice on day 0; CY + Tarin–CY-immunosuppressed mice treated with 200 μg tarin on day 0, 2 and 5; Tarin–mice treated with 200 μg tarin on days 0, 2, and 5; and Control–mice inoculated with saline. Results are expressed as means ± standard deviation of three independent experiments (n = 3). (TIFF) [file pone.0206240.s002.tiff]
